# Is silver diamine fluoride effective in reducing dentin hypersensitivity? A systematic review

**DOI:** 10.34172/joddd.2023.35449

**Published:** 2023-07-17

**Authors:** Érica Torres de Almeida Piovesan, Júlia Barros Alves, Caroline Diniz Pagani Vieira Ribeiro, Carla Massignan, Ana Cristina Barreto Bezerra, Soraya Coelho Leal

**Affiliations:** Department of Dentistry, School of Health Science, University of Brasilia (UnB), Brasilia, Brazil

**Keywords:** Silver diamine fluoride, Dentin hypersensitivity, Tooth sensitivity, Systematic review

## Abstract

**Background.:**

The purpose of this systematic review was to assess the clinical efficacy (sensitivity reduction) and safety (gum damage) of silver diamine fluoride (SDF) as a tooth desensitizer for adults.

**Methods.:**

The search strategy was developed and adapted from 12 databases. Two independent reviewers selected the studies in consensus with a third reviewer. Randomized clinical trials with adult volunteers affected by dentin hypersensitivity (DH), and receiving treatment with SDF were included. Studies with volunteers testing tooth whitening products, using some type of desensitizer, or taking analgesic or anti-inflammatory medication were excluded. The risk of bias was assessed according to the RoB 2 tool, and confidence in cumulative evidence, according to GRADE.

**Results.:**

Only 3 articles were included. The average pain assessed using the visual analog scale was lower in the SDF groups than in the short-term control groups (24h to 7 days) (*P*=0.0134 and *P*=0.0015) of the two studies. The third study evaluated a combination of SDF and a CO_2_ laser, compared to using only SDF, and found no statistical difference between the two (*P*=0.74). Inflammation and gingival staining were also evaluated in two of the three studies. No adverse effects were reported. All the included studies had a high risk of bias, and the certainty of the evidence was very low.

**Conclusion.:**

SDF can be used as a safe and effective tooth desensitizer in adults, with good results, as was achieved in a short-term follow-up. However, more studies with longer evaluation periods are required.

## Introduction

 Dentin hypersensitivity (DH) is an abnormal response of the tooth to mechanical, thermal, chemical, and osmotic stimuli, and is characterized by specific acute short-term pain.^1,2^ The prevalence of DH is highly heterogeneous, ranging between an estimated 3.8% to 85%. This is a common clinical problem, the incidence is believed to be increasing,^3^ and it is more frequent in patients suffering from periodontal disease. It most often occurs between 20 and 50 years of age, and is more common among women.^1^

 Difficulties in treating DH have led to a variety of therapeutic techniques and procedures for pain relief.^1^ Oxalates, calcium phosphate, fluoride solutions, sodium fluoride varnish, and gels have been shown to reduce sensitivity.^4^ However, there is continuing interest in finding effective treatments. A common desensitizing agent is silver diamine fluoride (SDF).^5^ It is effective in controlling caries progression, and can play a role in managing DH.^6^ SDF is an odorless, colorless alkaline solution containing silver ions and fluoride that form a complex with ammonia.

 SDF can easily be applied, and is very affordable. The product can be used outside the clinical environment, by those who are unable to tolerate invasive treatments, elderly populations, and those who are medically compromised, or have additional care and support needs. It is well accepted by children, as well.^7,8^ Therefore, SDF can be considered a user-friendly material for application in dental clinics, as well as remote areas, schools, or deprived communities.^9^

 There is yet no consensus on the mechanism that causes DH. However, Brannstrom’s hydrodynamic theory is currently the most widely accepted assumption put forth in the literature.^3^ The mechanism behind SDF and sensitivity control is that this aqueous silver and fluoride solution can produce a squamous layer over the exposed dentin, partially plugging the dentinal tubules from the exposed dentin, thereby reducing fluid shifts in the dentinal tubules.^10^ A series of chemical reactions takes place to promote tooth desensitization and carious lesion arrest by blocking dentinal tubules, promoting bacterial death, remineralizing the demineralized tooth, and inhibiting dentinal collagen degradation. These chemical reactions have the side effect of staining carious lesions (enamel and dentin) permanently black, but sound enamel does not stain.^6^

 The efficacy of SDF is documented in the literature, and is compared with that of negative control groups, and that of active treatments. Numerous systematic reviews have been undertaken to understand how SDF manages dental caries; however, the evidence remains uncertain and inconclusive regarding its use and treatment protocol as a desensitizer.

 The purpose of this systematic review was to assess the clinical efficacy and safety of SDF as a tooth desensitizer in adults by answering the following PICO (participant, intervention, comparator, and outcome) question: Is SDF effective in reducing DH?

## Materials and Methods

 The results of this systematic review were reported according to the Preferred Reporting Items for Systematic Reviews and Meta‐analyses (PRISMA).^11^

###  Eligibility criteria 

 Randomized controlled trials (RCTs) with adult volunteers ( ≥ 18 years) who presented with DH, and who received treatment with SDF were included. The eligibility criteria followed the Population, Intervention, Comparator, Outcome, and Study design (PICOS), where P was adults with DH; I was treatment with SDF; C was any comparator presented in the study, either placebo or hypersensitivity treatment; O was pain, and S was RCT.

 The exclusion criteria consisted of studies with volunteers who underwent tooth whitening in-home treatments using dentin desensitizers, like toothpaste or mouthwash, and studies with volunteers using analgesic or anti-inflammatory medication. In addition, prospective and retrospective cohort studies, case series, case reports, reviews, and full texts that were not found were also excluded. No date or language restrictions were applied. The intervention group was composed of adults who presented with DH, and who received treatment with SDF, while the control group consisted of placebo or other in-office desensitizing agents. Patients treated with SDF were considered the “intervention group,” regardless of whether they were the control or the case group in the primary studies.

 The primary outcome was DH, and the secondary outcome was related to safety, including any gingival or color changes caused by SDF.

###  Information sources, search strategy and selection process

 The search strategy for this review was conducted on March 10, 2021, in the following electronic databases: PubMed, Cochrane Central Register of Controlled Trials (CENTRAL) (Cochrane Library), EMBASE, Scopus, Web of Science, Livivo, LILACS (Portuguese and Spanish), Dentistry and Oral Sciences Source - DOSS (EBSCO), ProQuest and Open Grey. The Google Scholar search was conducted on April 5, 2021. Additionally, a manual search of the reference lists of all the eligible studies was performed. The search strategy is detailed in Supplementary File 1.

 The MeSH (PubMed), Emtree (EMBASE), and DeCS (LILACS) controlled vocabularies were used to develop the search strategies. The same terms and descriptors were used on all search strategies (customized according to the specificities of the database) to confirm the search consistency. The terms were exploded whenever appropriate, to ensure that relevant studies would not be missed. A wide variety of free terms were included to provide a comprehensive search. All the references were managed with the EndNote (Clarivate Analytics, USA) software program, and duplicate studies were removed.

 The reading was done in two phases, and by two authors independently. The first phase consisted of reading the titles and abstracts using the Rayyan online software program. In the second phase, the full-text version of every potentially relevant study was obtained and reviewed by the same researchers, based on the inclusion and exclusion criteria. All disagreements were resolved by consensus with a third author.

###  Data collection process and items

 Two previously trained and independent authors collected data from the included studies using a pre-established data collection form. Disagreements were resolved by discussing with a third reviewer. A specific data extraction matrix was created to collect information from each study being analyzed, including the study author, year, country of origin and characteristics, as well as the characteristics of the participants, intervention description, comparison group, outcome measures, and major findings. The descriptive characteristics of the studies were categorized manually.

###  Study risk of bias assessment

 The risk of bias was assessed using the RoB 2 tool.^12^ The RoB 2 was used independently by two reviewers, who came to a consensus to determine the risk of bias. The five domains evaluated by RoB 2 are bias arising from the randomization process; deviations from intended interventions; missing outcome data; measurement of the outcome; and selection of the reported result. The overall risk of bias is classified based on the answers to signaling questions, and can range from low risk, some concerns and high risk of bias.

###  Statistical analysis 

 A meta-analysis was planned, but could not be conducted, because of methodological differences regarding the interventions in the design. Even though the same concentration of SDF was used, different brands were also used. Accordingly, a descriptive analysis was preferred.

###  Reporting bias assessment

 The study records were checked. The reporting bias was evaluated primarily based on the comparison involving projected results and statistical analysis plans and methods with the reported results. When available, the registered protocols of the included studies were searched to compare the planned with the reported outcomes. Since a meta-analysis was not performed, the funnel plot was not used to assess the effects of small studies. A large bibliographic search was also made to avoid the publishing bias, in addition to the search for the sponsors of each study included to determine whether there was any conflict of interest by the authors.

###  Certainty assessment

 The Grading of Recommendations Assessment, Development, and Evaluation (GRADE) method was used to appraise the evidence that emerged from this review.^13^

## Results

###  Study selection 

 In phase one, 422 studies were identified across all the selected electronic databases, and 388 studies were chosen using supplementary searches (grey literature), totaling 810 studies. After the duplicates were removed, the remaining 532 titles and abstracts were evaluated. Only 7 articles were included in phase two, according to the eligibility criteria.

**Figure 1:**
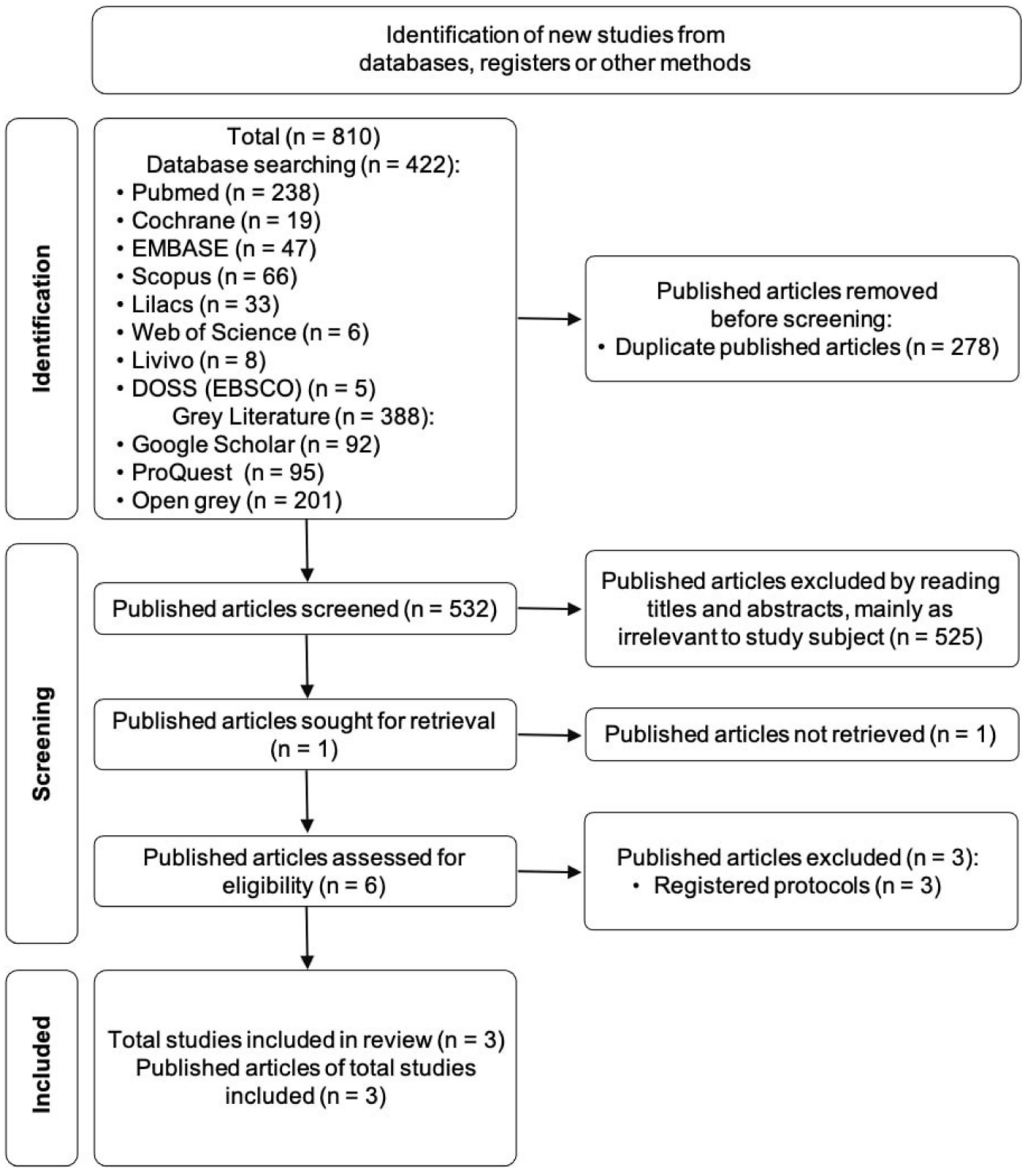
Flowchart adapted from PRISMA

 In phase two, 3 of the studies were excluded because they were registered protocols, and the results were not yet available.^14-16^ Another study was excluded because its full text was not made available,^17^ even after attempting to contact the author of the study by email. The reasons for excluding each of the studies are described in Supplementary File 2, and the selection of the studies is presented as a flow diagram (Figure 1).

###  Study characteristics 

 One of the 3 clinical trials included was conducted in the US and published in 2011,^4^ one in Australia and published in 2012,^18^ and the other, in Thailand and published in 2018.^19^ The sample size ranged from 16 to 126 participants older than 18 years of age. The 3 clinical trials were randomized; one compared two parallel groups,^4^ one was double-blind with split-mouth,^18^ and the third was single-blind with split-mouth.^19^

 The selected studies sought to evaluate the reduction in pain caused by tooth sensitivity from the application of the SDF. All the studies used the visual analog scale (VAS) to assess the primary outcome, which was pain,^4,18,19^ and one of them also used DIAGNOdent.^18^ Regarding the use of the VAS, there was a difference among the articles, namely, the scale used in one of the studies was 100 mm,^4^ and that used in the other two was 1 to 10 mm.^18,19^ Only 2 studies evaluated secondary outcomes, and both reported side effects in the gingival tissues.^4,18^ Table 1 offers an overview of the three studies included, and the main data extracted.

**Table 1:** Summary of characteristics of the included studies

**Author, year; country**	**Primary outcome - Hypersensitivity**	**Findings of the primary outcome in the control group**	**Findings of the primary outcomes in the intervention group - SDF**	**Secondary outcomes (safety)**	**Findings of secondary outcomes in the control group**	**Findings of secondary outcomes in the intervention group - SDF**	**Main conclusion**	**Summary**
Castillo etal, ^4^ 2011; Peru	Reduction in pain (tooth sensitivity) VAS.	Sterile water: Mean (SD) [range] Lima: - Baseline (B): 49.3 (19.3) [15, 84] - 24h: 52.1 (22.8) [16, 89] - B changes (C): 2.6 (15.3) [-44, 32] - 7d: 49.9 (21.2) [9, 85] - BC: 0.4 (16.2) [-38, 33] Cusco: - B: 51.6 (22.4) [16, 99] - 24h: 50.6 (22.0) [15, 95] - BC: -1.0 (11.7) [-37, 20] - 7d: 46.1 (24.4) [3, 92] - BC: -5.5 (18.1) [-77, 18]	Mean (SD) [range] Lima: - B: 57.3 (26.7) [17, 99] - 24h: 28.2 (22.1) [2, 75] - BC: -29.1 (27.5) [-94, 10] - 7d: 21.5 (23.0) [1, 78] - BC: -35.8 (27.7) [-97, 12] Cusco: - B: 51.7 (20.5) [22, 92] - 24h 45.2 (24.1) [11, 87] - BC: 6.5 (13.1) [-34, 22] - 7d: 28.3 (21.8) [2, 94] - BC: -23.4 (21.0) -23.4 (21.0)	Erythema, gingival inflammation and ulceration. Assessed visually (1-3) and changes present or absent.	Sites combined (n = 63) - B: 9 (14.3) - 24h: 4 (6.3) - 7d: 4 (6.3	Sites combined (n = 63) - B: 9 (14.3) - 24h: 13 (20.6) - 7d: 6 (9.5)	SDF is clinically effective and safe as a tooth desensitizer after 24h and 7d. Staining of teeth was found only when surfaces had untreated decay. The inclusion of decayed teeth was not mentioned, and this can be confusing regarding the outcome.	+
Craig et al,^18^ 2012; Australia	Reduction in dentine hypersensitivity. VAS	Oxalic acid-based preparation: Mean (SD) - B: 6.87 [1.93] - 7d: 6.18 [2.16] - BC: - 0.69 [1.55] Participants’ subjective assessment of changes over 7d: 2	SDF + potassium iodide product: Mean (SD) - B: 7.54 [1.54] - 7d: 5.83 [2.08] - BC: - 1.71 [1.62] Participants’ subjective assessment of changes over 7d: 12	Adverse side effects to gingival tissues or staining of teeth surfaces. Changes in inflammation or other gingival changes. Clinical photographs	There were no observable changes in inflammation or other changes in the gingival tissues and no staining was observed on any of the teeth.	Not reported	The SDF + potassium iodide produced a significantly larger reduction in dentine hypersensitivity than oxalic acid preparation.	+
Permata et al,^19^ 2018; Indonesia	Reduction in DH score. VAS(evaporative and thermal) DIAGNOdent	SDF + CO2 laser: The *P* value (Mean/ Range/ Median) VAS for evaporative stimulus: -B: 0,91 (2.41/0–9/2) - Immediate: 0.27 (0.67/0–5/0) - 7d: 0.95 (0.59/0–4/0) - 14d: 0.74 (0.47/0-4/0) VAS for thermal stimulus: - B: 0.59 (3.20/0–8/3) - Immediate: 0.36(1.04/0–8/0) - 7d:0.10 (0.59/0–5/0) - 14d:0.31 (0.71/0–6/0) DIAGNOdent: - B:0.53 (8.73/1-41/7) - Immediate: 0.35(4.02/0-19/2) - 7d:0.90 (5.00/1-21/3_ -14d:0.28 (4.69/0-22/3)	SDF: The *P* value (Mean/Range/Median) VAS for evaporative stimulus: - B: 0,91(2.45/0–9/1.5) - Immediate: 0,27(0.83/0–9/0 - 7d: 0.95 (1.02/0–8/0) - 14d: 0.74 (1.0/ 0-10 0) VAS for thermal stimulus - B: 0.59(3.45/0–9/3) - Immediate: 0.36(1.36/0–8/1) - 7d:0.10 (1.05/0-7is/0) - 14d:0.31 (0.95/0–9/0) DIAGNOdent: -B: 0.53 (10.31/0-48/7.5) - Immediate: 0.35(6.21/0-32/3) - 7d: 0.90 (5.71/0-25/3.5) - 14d: 0.28 (5.52/0-18/4)	The study had no secondary outcome.	The study had no secondary outcome.	The study had no secondary outcome.	SDF used alone or in combination with CO2 laser treatment can reduce DH. Decrease in VAS scores was greater for teeth treated with SDF and CO2 laser than for teeth treated only with SDF with no statistical difference between the two.	=

Abbreviations: SDF, silver diamine fluoride; DH, dentin hypersensitivity. Note: ( + ) improvement in the hypersensitivity of the intervention group; ( - ) improvement in the control group ( = ) No difference.

 In two studies, the inclusion criteria were similar for adult patients with at least two teeth in the upper arch displaying symptoms of DH.^18,19^ One of the studies was carried out comparatively between two groups; hence, the inclusion criterion had at least one vital cusp or premolar with a cervical vestibular defect and clinical hypersensitivity.^4^

 All the studies that recruited participants who were using some type of dental desensitizer, prescription drugs, non-steroidal anti-inflammatory drugs, or aspirin, and pregnant women were excluded from participating.^4,18,19^ Other exclusion criteria presented in other studies were participants who had received a fluoride varnish treatment in the previous month,^18,19^ who used smokeless tobacco or chewed coca leaves, or who were sensitive to silver or other heavy metal ions.^4,19^

###  Risk of bias in individual studies

**Figure 2:**
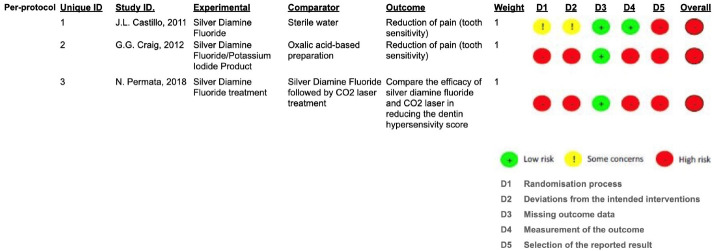
ROB 2 - risk of bias

 All clinical trials included were at a high risk of bias, according to the RoB 2 assessment shown in Figure 2, and described in Supplementary file 3. The determinants for increasing the risk of bias were the randomization process, the concealment of allocation, the blinding method, when proposed, and the reported results. The lack of outcomes was the determinant that least influenced the risk of bias.

###  Results of individual studies 

 Two studies evaluated whether or not SDF is clinically effective both 24 hours and 7 days after application^4,18^; another study evaluated the same periods, as well as 14 days later, and also compared SDF in combination with a CO_2_ laser.^19^ One of the studies indicated that males were more commonly affected by DH.^19^

 Castillo et alcarried out a study in two different locations with 126 adults (mean age 43.5 years) affected by tooth sensitivity; the participants in each location were divided into two parallel groups. One group received 38% SDF (Saforide, Osaka, Japan), and the other was the control group (sterile water). The group receiving SDF treatment had an average VAS score of 57.3, declining to 21.5 after 7 days. In the second location, the group treated with SDF had an average VAS score of 51.7, dropping to 28.3 after 7 days. Thus, it was demonstrated that SDF was effective in reducing DH-related pain. In the control groups of both first and second locations, the mean of the initial and final VAS scores remained stable after 7 days (*P* = 0.0015).^4^

 A study performed by Craig et alwith 19 adults (mean age 38.7 years) affected by tooth sensitivity, reported that the average difference between VAS scores immediately before and 7 days after the application of 38% SDF (Riva Star, SDI Limited, Melbourne, Australia) was greater than that of teeth treated with oxalic acid-based preparation within the same time frame. VAS at baseline was 7.54 and at 7 days was 5.83 for teeth treated with SDF/potassium iodide, and VAS at baseline was 6.87 and at 7 days was 6.18 for teeth treated with oxalic. The mean difference between VAS at baseline and at 7 days for teeth treated with SDF/potassium iodide was 1.71, compared with 0.69 for teeth treated with oxalic acid (*P* = 0.0134).^18^

 Permata *et al *conducted a study with 16 adults (mean age of 25.5 years) comparing the use of 38% SDF (Saforide, Osaka, Japan) in one group versus SDF combined with a CO_2_ laser in another group. Initially, the SDF group had a mean pre-application VAS score of 7.83, decreasing to 5.67 14 days post-application, demonstrating that there was a reduction in pain in a short period of time. In the SDF + CO_2_ laser group, the mean VAS score initially was 7.33, and dropped to 1.83 (*P* = 0.74) after 14 days of application,^19^ indicating that there was no statistical difference between SDF alone and SDF combined with a CO_2_ laser.

 Regarding gum damage caused by SDF application, the two studies that evaluated this secondary outcome observed that there was no adverse effect resulting from SDF application. If there were any alterations, they were momentary and mild.^4,19^

###  Results of syntheses 

 All the studies included in this systematic review tested SDF to assess pain reduction related to DH, and one of these also evaluated SDF combined with a CO_2_ laser. The results of all the studies confirmed that SDF has a short-term effect (24 hours to 14 days) on DH.^4,18,19^

###  Reporting bias

 No reporting bias was found after analyzing the methods and results of the studies included; all the measures taken were reported, whether or not they were statistically significant. However, only one of the three studies reported the protocol registration number^4^; hence, the studies could not be evaluated as to whether they were conducted as planned.

###  Certainty of the levels of evidence 

 The certainty in cumulative evidence was very low, according to GRADE.^13^ The randomized clinical trials were evaluated for risk of bias, inconsistency, indirectness, and imprecision. The outcomes of reduction in pain, gingival inflammation, and color change contributed to this decision. The explanations for the assessment of certainty in cumulative evidence are presented in Table 2.

**Table 2:** Certainty of evidence assessment based on GRADE

**Certainty assessment**							**Impact**	**Certainty**	**Importance**
No. of studies	Study design	Risk of bias	Inconsistency	Indirectness	Imprecision	Other considerations			
**Reduction of pain (tooth sensitivity)**									
3	Randomized trials	Very serious^a^	Very serious^b^	Serious^c^	Very serious^d^	None		⨁◯◯◯ Very Low	
**Gingival inflammation (assessed with: assessed visually)**									
3	Randomized trials	Very serious^a^	Very serious^e^	Very serious^f^	Very serious^g^	None		⨁◯◯◯ Very Low	
**Color changes (assessed with: changes - present or absent)**									
3	Randomized trials	Very serious^a^	Very serious^h^	Very serious^f^	Very serious^g^	None		⨁◯◯◯ Very Low	

CI: Confidence interval ^a^Most studies did not explain how randomization and blinding were done. ^b^The studies were heterogeneous. The methodology among the studies was different. All studies used the VAS scale, but one of the studies used the 100mm scale, and the other two used from 1 to 10mm. The number of participants was very different among the studies. ^c^All studies contemplate the PICO strategy. However, the control groups use different desensitizing agents. ^d^There was no sample calculation. Two of the three studies had a very small sample. ^e^There was no standardization for the evaluation of gingival inflammation. ^f^The interventionists knew the groups, and this can influence the visual judgment. ^g^Two studies did not use a reliable scale, and one did not evaluate the color change or gingival inflammation. ^h^There was no standardization for the color change.

## Discussion

 To our knowledge, this study is the first systematic review on the clinical efficacy and safety of SDF as a tooth desensitizer for adults. Although SDF has been licensed for managing DH, there are just a few trials and no systematic reviews on this topic.

 The data from the primary studies in our review support the finding that SDF at a 38% concentration has the potential to reduce sensitivity. However, two of the studies^18,19^ included only 19 and 16 subjects respectively; hence, the reliability of these studies must be questioned. The other study^4^ evaluated 126 adults with sensitivity, and found that SDF was able to significantly reduce sensitivity among adult patients both 24 hours and 7 days post-application. Unfortunately, none of the studies evaluated the effect of SDF for longer periods. Although the findings seem promising, longer evaluation periods and comparisons with alternate sensitivity treatments are required to gain a better understanding of its efficacy.

 Regarding the safety of SDF applied as a sensitivity agent, the two studies^4,18^ that evaluated gum damage observed that SDF application caused no adverse effect. Another issue concerning SDF in comparison with alternative sensitivity agents is the possible formation of silver black spots. However, a study^4^ found that staining occurred only when there was caries on the surface of the exposed dentin.

 Several limitations could be observed in this study. One of the concerns about conducting a systematic review with a small number of RCTs is the risk of bias. The quality of the evidence produced in the present review was graded as very low, since the evidence came from RCTs with a high risk of bias.^13^ All the clinical trials included were at a high risk of bias, which aroused great concern regarding the reliability of these studies. The determinants for increasing the risk of bias were the randomization process, the concealment of allocation, the blinding method, when proposed, and the reported results. The lack of outcomes was the determinant that least influenced the risk of bias.

 Furthermore, there was considerable heterogeneity in the included studies, such as the overall mean age of the participants, the size of the samples, and the duration of the interventions. The efficacy of SDF could not be statistically analyzed, and that of the SDF combination with CO_2_ not be assessed either, because of the variety of products included in the studies (Saforide, Osaka, Japan and Riva Star, SDI Limited, Melbourne, Australia).

 All the studies clearly described the procedures used to assess the primary outcome, and VAS was used to assess pain, because it is a method that allows replication. However, longitudinal studies that follow up large study populations over a longer period are still greatly needed, and are of utmost importance to increase the reliability of results provided by substantial sample sizes.

 Based on the quality assessment of the 3 studies, implications for future avenues of investigation were identified. Further research is needed to develop a definitive protocol that allows better comparison among the results available in the literature, and greater understanding of the exact mechanism of action of SDF in DH.

## Conclusion

 Within the limitations of this systematic review, it is possible to infer that SDF (38%), whether or not combined with other therapies, has shown beneficial effects in the treatment of DH, and is considered safe and effective. Nevertheless, more studies with longer evaluation periods are required to gain a better understanding regarding SDF efficacy in the long term.

## Competing Interests

 All the authors declare that they have no conflict of interest, and confirm that this paper has not been published previously, or submitted in any other language, and is not being considered for publication elsewhere.

## Ethical Approval

 The protocol of this systematic review has been published and registered at the International Prospective Register of Systematic Reviews (PROSPERO) under registration number: CRD42018107102. No changes were made to the protocol during this systematic review.

## Funding

 This study was financed in part by the Coordenação de Aperfeiçoamento de Pessoal de Nível Superior – Brasil (CAPES).

## Supplementary Files

Supplementary file 1. Database search strategy.

Supplementary file 2. Excluded articles and reasons for exclusion.

Supplementary file 3. ROB 2 - Risk of Bias.
