# Supplementary material for: Is silver diamine fluoride effective in reducing dentin hypersensitivity? A systematic review
Source: J Dent Res Dent Clin Dent Prospects. 2023 Jul 17;17(2):63–70. doi: 10.34172/joddd.2023.35449 (PMC10462467; doi:10.34172/joddd.2023.35449)
Supplement: Supplementary file 1 — Database search strategy. [file joddd-17-63-s001.pdf]

Piovesan et al, *J Dent Res Dent Clin Dent Prospects,* 2023, 17(2), 63-70
doi: 10.34172/joddd.2023.35449
https://joddd.tbzmed.ac.ir

Supplementary file 1. Database search strategy

**Database**	**Search (March 10th)**
**PubMed**	(("Dentin Desensitizing Agents"[Mesh] OR "Dentin Desensitizing Agents" OR "Desensitizing Agents Dentin" OR "desensitise" OR "desensitised" OR "desensitiser" OR "desensitises" OR "desensitising" OR "Sensodyne Dentin Desensitizer" [Supplementary Concept] OR "gluma desensitizer" [Supplementary Concept] OR "Gluma Dentin Desensitizer" OR "Dentin desensitizers" OR "UltraEZ" [Supplementary Concept] OR "SuperSeal" [Supplementary Concept] OR "D-Sense 2" [Supplementary Concept] OR "oxa-gel" [Supplementary Concept] OR "Pain-Free" [Supplementary Concept] OR "DentinBloc" [Supplementary Concept] OR "Micro PrimeTM" [Supplementary Concept]) AND ("Silver Diamine Fluoride" [Supplementary Concept] OR "Silver Diamine Fluoride" OR "Diamine Silver Fluoride" OR "Silver Ammonia Fluoride" OR "Silver Diammine Fluoride" OR "Diammine Silver Fluoride" OR "Silver Fluoride" [Supplementary Concept] OR "Cariostatic Agents"[Mesh] OR "Cariostatic Agents" OR "Cariostatic" OR "Silver Compounds"[Mesh] OR "Silver Compounds" OR "SDF"[Title/Abstract])) AND ("hypersensitivity"[MeSH Terms] OR "hypersensitivity"[Title/Abstract] OR "Hypersensitivities"[Title/Abstract] OR "Dentin Sensitivity"[Mesh] OR "Dentin Sensitivity" OR "Dentin Sensitivities" OR "Dentine Hypersensitivity" OR "Dentin Hypersensitivities" OR "Dentine Sensitivity" OR "Tooth Sensitivity" OR "Dentin Hypersensitivity" OR "Dentin"[Mesh] OR "Dentin" OR "Dentine" OR "Dentines" OR "Dentinal" OR "Dentinal Sensitivity" OR "Tooth"[Mesh] OR "Tooth" OR "Teeth" OR "Sensitivity"[Title/Abstract] OR "Sensitivities"[Title/Abstract])
**Cochrane**	("Dentin Desensitizing Agents" OR "Desensitizing Agents Dentin" OR "desensitise" OR "desensitised" OR "desensitiser" OR "desensitises" OR "desensitising" OR "Sensodyne Dentin Desensitizer" OR "gluma desensitizer" OR "Gluma Dentin Desensitizer" OR "Dentin desensitizers" OR "UltraEZ" OR "SuperSeal" OR "D-Sense 2" OR "oxa-gel" OR "Pain-Free" OR "DentinBloc" OR "Micro PrimeTM") AND ("Silver Diamine Fluoride" OR "Diamine Silver Fluoride" OR "Silver Ammonia Fluoride" OR "Silver Diammine Fluoride" OR "Diammine Silver Fluoride" OR "Silver Fluoride" OR "Cariostatic Agent" OR "Cariostatic Agents" OR "Cariostatic" OR "Silver Compounds" OR "SDF") AND ("hypersensitivity" OR "Hypersensitivities" OR "Dentin Sensitivity" OR "Dentin Sensitivities" OR "Dentine Hypersensitivity" OR "Dentin Hypersensitivities" OR "Dentine Sensitivity" OR "Tooth Sensitivity" OR "Dentin Hypersensitivity" OR "Dentin" OR "Dentin" OR "Dentine" OR "Dentines" OR "Dentinal" OR "Dentinal Sensitivity" OR "Tooth" OR "Teeth" OR "Sensitivity" OR "Sensitivities") in Title Abstract Keyword - (Word variations have been searched)

**EMBASE**	('dentin desensitizing agents'/exp OR 'dentin desensitizing agents' OR 'desensitizing agents dentin' OR 'desensitise' OR 'desensitised' OR 'desensitiser' OR 'desensitises' OR 'desensitising' OR 'sensodyne dentin desensitizer' OR 'gluma desensitizer'/exp OR 'gluma desensitizer' OR 'gluma dentin desensitizer' OR 'dentin desensitizers' OR 'ultraez' OR 'superseal'/exp OR 'superseal' OR 'd-sense 2' OR 'oxa-gel' OR 'pain-free' OR 'dentinbloc' OR 'micro primetm') AND ('silver diamine fluoride'/exp OR 'silver diamine fluoride' OR 'diamine silver fluoride' OR 'silver ammonia fluoride' OR 'silver diammine fluoride' OR 'diammine silver fluoride' OR 'silver fluoride'/exp OR 'silver fluoride' OR 'cariostatic agent'/exp OR 'cariostatic agent' OR 'cariostatic agents'/exp OR 'cariostatic agents' OR 'cariostatic' OR 'silver compounds'/exp OR 'silver compounds' OR 'sdf') AND ('hypersensitivity'/exp OR 'hypersensitivity' OR 'hypersensitivities' OR 'dentin sensitivity'/exp OR 'dentin sensitivity' OR 'dentin sensitivities' OR 'dentine hypersensitivity'/exp OR 'dentine hypersensitivity' OR 'dentin hypersensitivities' OR 'dentine sensitivity' OR 'tooth sensitivity'/exp OR 'tooth sensitivity' OR 'dentin hypersensitivity'/exp OR 'dentin hypersensitivity' OR 'dentin'/exp OR 'dentin' OR 'dentine'/exp OR 'dentine' OR 'dentines' OR 'dentinal' OR 'dentinal sensitivity' OR 'tooth'/exp OR 'tooth' OR 'teeth'/exp OR 'teeth' OR 'sensitivity'/exp OR 'sensitivity' OR 'sensitivities')
**Scopus**	TITLE-ABS-KEY (("Dentin Desensitizing Agents" OR "Desensitizing Agents Dentin" OR "desensitise" OR "desensitised" OR "desensitiser" OR "desensitises" OR "desensitising" OR "Sensodyne Dentin Desensitizer" OR "gluma desensitizer" OR "Gluma Dentin Desensitizer" OR "Dentin desensitizers" OR "UltraEZ" OR "SuperSeal" OR "D-Sense 2" OR "oxa-gel" OR "Pain-Free" OR "DentinBloc" OR "Micro PrimeTM") AND ("Silver Diamine Fluoride" OR "Diamine Silver Fluoride" OR "Silver Ammonia Fluoride" OR "Silver Diammine Fluoride" OR "Diammine Silver Fluoride" OR "Silver Fluoride" OR "Cariostatic Agent" OR "Cariostatic Agents" OR "Cariostatic" OR "Silver Compounds" OR "SDF") AND ("hypersensitivity" OR "Hypersensitivities" OR "Dentin Sensitivity" OR "Dentin Sensitivities" OR "Dentine Hypersensitivity" OR "Dentin Hypersensitivities" OR "Dentine Sensitivity" OR "Tooth Sensitivity" OR "Dentin Hypersensitivity" OR "Dentin" OR "Dentin" OR "Dentine" OR "Dentines" OR "Dentinal" OR "Dentinal Sensitivity" OR "Tooth" OR "Teeth" OR "Sensitivity" OR "Sensitivities"))

**Web of Science**	TS=(("Dentin Desensitizing Agents" OR "Desensitizing Agents Dentin" OR "desensitise" OR "desensitised" OR "desensitiser" OR "desensitises" OR "desensitising" OR "Sensodyne Dentin Desensitizer" OR "gluma desensitizer" OR "Gluma Dentin Desensitizer" OR "Dentin desensitizers" OR "UltraEZ" OR "SuperSeal" OR "D-Sense 2" OR "oxa-gel" OR "Pain-Free" OR "DentinBloc" OR "Micro PrimeTM") AND ("Silver Diamine Fluoride" OR "Diamine Silver Fluoride" OR "Silver Ammonia Fluoride" OR "Silver Diammine Fluoride" OR "Diammine Silver Fluoride" OR "Silver Fluoride" OR "Cariostatic Agent" OR "Cariostatic Agents" OR "Cariostatic" OR "Silver Compounds" OR "SDF") AND ("hypersensitivity" OR "Hypersensitivities" OR "Dentin Sensitivity" OR "Dentin Sensitivities" OR "Dentine Hypersensitivity" OR "Dentin Hypersensitivities" OR "Dentine Sensitivity" OR "Tooth Sensitivity" OR "Dentin Hypersensitivity" OR "Dentin" OR "Dentin" OR "Dentine" OR "Dentines" OR "Dentinal" OR "Dentinal Sensitivity" OR "Tooth" OR "Teeth" OR "Sensitivity" OR "Sensitivities"))
**Livivo**	"Diamine Silver Fluoride" AND Sensitivity
**Lilacs (Portuguese and Spanish)**	("Diamine Silver Fluoride" OR "Cariostatic" OR "Cariostáticos" OR "Cariostático" OR "Dessensibilizantes Dentinários" OR "Desensibilizante Dentinario") AND ("Dentin Hypersensitivities" OR "Dentine Sensitivity" OR "Sensibilidade da Dentina" OR "Sensibilidad de la Dentina") AND (db:("LILACS"))
**Dentistry and Oral Sciences Source - DOSS (EBSCO)**	("Dentin Desensitizing Agents" OR "Desensitizing Agents Dentin" OR "desensitise" OR "desensitised" OR "desensitiser" OR "desensitises" OR "desensitising" OR "Sensodyne Dentin Desensitizer" OR "gluma desensitizer" OR "Gluma Dentin Desensitizer" OR "Dentin desensitizers" OR "UltraEZ" OR "SuperSeal" OR "D-Sense 2" OR "oxa-gel" OR "Pain-Free" OR "DentinBloc" OR "Micro PrimeTM") AND ("Silver Diamine Fluoride" OR "Diamine Silver Fluoride" OR "Silver Ammonia Fluoride" OR "Silver Diammine Fluoride" OR "Diammine Silver Fluoride" OR "Silver Fluoride" OR "Cariostatic Agent" OR "Cariostatic Agents" OR "Cariostatic" OR "Silver Compounds" OR "SDF") AND ("hypersensitivity" OR "Hypersensitivities" OR "Dentin Sensitivity" OR "Dentin Sensitivities" OR "Dentine Hypersensitivity" OR "Dentin Hypersensitivities" OR "Dentine Sensitivity" OR "Tooth Sensitivity" OR "Dentin Hypersensitivity" OR "Dentin" OR "Dentin" OR "Dentine" OR "Dentines" OR "Dentinal" OR "Dentinal Sensitivity" OR "Tooth" OR "Teeth" OR "Sensitivity" OR "Sensitivities")

**ProQuest**	("Dentin Desensitizing Agents" OR "Desensitizing Agents Dentin" OR "desensitise" OR "desensitised" OR "desensitiser" OR "desensitises" OR "desensitising" OR "Sensodyne Dentin Desensitizer" OR "gluma desensitizer" OR "Gluma Dentin Desensitizer" OR "Dentin desensitizers" OR "UltraEZ" OR "SuperSeal" OR "D-Sense 2" OR "oxa-gel" OR "Pain-Free" OR "DentinBloc" OR "Micro PrimeTM") AND ("Silver Diamine Fluoride" OR "Diamine Silver Fluoride" OR "Silver Ammonia Fluoride" OR "Silver Diammine Fluoride" OR "Diammine Silver Fluoride" OR "Silver Fluoride" OR "Cariostatic Agent" OR "Cariostatic Agents" OR "Cariostatic" OR "Silver Compounds" OR "SDF") AND ("hypersensitivity" OR "Hypersensitivities" OR "Dentin Sensitivity" OR "Dentin Sensitivities" OR "Dentine Hypersensitivity" OR "Dentin Hypersensitivities" OR "Dentine Sensitivity" OR "Tooth Sensitivity" OR "Dentin Hypersensitivity" OR "Dentin" OR "Dentin" OR "Dentine" OR "Dentines" OR "Dentinal" OR "Dentinal Sensitivity" OR "Tooth" OR "Teeth" OR "Sensitivity" OR "Sensitivities")
**Open Grey**	("Dentin Desensitizing Agents" OR "Desensitizing Agents Dentin" OR "desensitise" OR "desensitised" OR "desensitiser" OR "desensitises" OR "desensitising" OR "Sensodyne Dentin Desensitizer" OR "gluma desensitizer" OR "Gluma Dentin Desensitizer" OR "Dentin desensitizers" OR "UltraEZ" OR "SuperSeal" OR "D-Sense 2" OR "oxa-gel" OR "Pain-Free" OR "DentinBloc" OR "Micro PrimeTM") AND ("Silver Diamine Fluoride" OR "Diamine Silver Fluoride" OR "Silver Ammonia Fluoride" OR "Silver Diammine Fluoride" OR "Diammine Silver Fluoride" OR "Silver Fluoride" OR "Cariostatic Agents" OR "Cariostatic Agents" OR "Cariostatic") AND ("Dentin Sensitivity" OR "Dentin Sensitivities" OR "Dentine Hypersensitivity" OR "Dentin Hypersensitivities" OR "Dentine Sensitivity" OR "Tooth Sensitivity" OR "Dentin Hypersensitivity" OR "Dentin" OR "Dentin" OR "Dentine" OR "Dentines")
**Google Scholar**	Allintitle:("desensitizing agents" OR cariostatic OR "silver diamine fluoride") AND (dentin OR tooth OR teeth)
