# Supplementary material for: Is silver diamine fluoride effective in reducing dentin hypersensitivity? A systematic review
Source: J Dent Res Dent Clin Dent Prospects. 2023 Jul 17;17(2):63–70. doi: 10.34172/joddd.2023.35449 (PMC10462467; doi:10.34172/joddd.2023.35449)
Supplement: Supplementary file 2 — Excluded articles and reasons for exclusion. [file joddd-17-63-s002.pdf]

Piovesan et al, *J Dent Res Dent Clin Dent Prospects,* 2023, 17(2), 63-70
doi: 10.34172/joddd.2023.35449
https://joddd.tbzmed.ac.ir

Supplementary file 2. Excluded articles and reasons for exclusion (n=4)

**Title**	**Reason for exclusion**
Effects of Diammine Silver Fluoride on Tooth Sensitivity (2010)[14]	1
Effects of desensitizing agents for cervical dentin hypersensitivity: A randomized clinical trial (2017)[15]	2
Efficacy of two professionally applied desensitizing agent (2018)[16]	1
Effect of desensitizing agents in reducing the dentinal hypersensitivity following vital tooth preparation (2020)[17]	1

1) Registration Protocol (n=3)
2) Full text not found (n=1)
