# Supplementary material for: Is silver diamine fluoride effective in reducing dentin hypersensitivity? A systematic review
Source: J Dent Res Dent Clin Dent Prospects. 2023 Jul 17;17(2):63–70. doi: 10.34172/joddd.2023.35449 (PMC10462467; doi:10.34172/joddd.2023.35449)
Supplement: Supplementary file 3 — ROB 2 - Risk of Bias. [file joddd-17-63-s003.pdf]

Piovesan et al, *J Dent Res Dent Clin Dent Prospects,* 2023, 17(2), 63-70
doi: 10.34172/joddd.2023.35449
https://joddd.tbzmed.ac.ir

Supplementary file 3. ROB 2 - Risk of Bias

**Unique ID**	1	**Study ID**	J. L. Castillo, 2011	**Assessor**	R1/R2
**Ref or Label**		**Aim**	Adhering to intervention (the 'per-protocol' effect)	**The effect of adhering to intervention…**	Non-adherence of trial participants to their assigned intervention
**Experimental**	Silver Diamine Fluoride	**Comparator**	Sterile water	**Source**	Journal article(s)
**Outcome**	Reduction of pain (tooth sensitivity)	**Results**		**Weight**	1

**Domain**	**Signaling question**	**Response**	**Comments**
**Bias arising from the randomization process**	1.1 Was the allocation sequence random?	Y	No information on allocation method
	1.2 Was the allocation sequence concealed until participants were enrolled and assigned to interventions?	NI	
	1.3 Did baseline differences between intervention groups suggest a problem with the randomization process?	N	
	**Risk of bias judgement**	**Some concerns**	
**Bias due to deviations from intended interventions**	2.1 Were participants aware of their assigned intervention during the trial?	N	
	2.2 Were carers and people delivering the interventions aware of participants' assigned intervention during the trial?	PN	
	2.3. [If applicable:] If Y/PY/NI to 2.1 or 2.2: Were important non-protocol interventions balanced across intervention groups?	NA	

	2.4. [If applicable:] Were there failures in implementing the intervention that could have affected the outcome?	NA	
	2.5. [If applicable:] Was there non-adherence to the assigned intervention regimen that could have affected participants' outcomes?	NI	No information on non-adherence to intervention
	2.6. If N/PN/NI to 2.3, or Y/PY/NI to 2.4 or 2.5: Was an appropriate analysis used to estimate the effect of adhering to the intervention?	Y	
	**Risk of bias judgement**	**Some concerns**	
**Bias due to missing outcome data**	3.1 Were data for this outcome available for all, or nearly all participants randomized?	Y	
	3.2 If N/PN/NI to 3.1: Is there evidence that the result was not biased by missing outcome data?	NA	
	3.3 If N/PN to 3.2: Could missingness in the outcome depend on its true value?	NA	
	3.4 If Y/PY/NI to 3.3: Is it likely that missingness in the outcome depended on its true value?	NA	
	**Risk of bias judgement**	**Low**	
**Bias in measurement of the outcome**	4.1 Was the method of measuring the outcome inappropriate?	N	
	4.2 Could measurement or ascertainment of the outcome have differed between intervention groups?	N	
	4.3 Were outcome assessors aware of the intervention received by study participants?	PN	
	4.4 If Y/PY/NI to 4.3: Could assessment of the outcome have been influenced by knowledge of intervention received?	NA	
	4.5 If Y/PY/NI to 4.4: Is it likely that assessment of the outcome was influenced by knowledge of intervention received?	NA	
	**Risk of bias judgement**	**Low**	
**Bias in selection of the reported result**	5.1 Were the data that produced this result analysed in accordance with a pre-specified analysis plan that was finalized before unblinded outcome data were available for analysis?	NI	Data don't make it clear
	5.2 ... multiple eligible outcome measurements (e.g. scales, definitions, time points) within the outcome domain?	PY	Data used VAS score and visual changes

	5.3 ... multiple eligible analyses of the data?			PN	
	**Risk of bias judgement**			**High**	
**Overall bias**	**Risk of bias judgement**			**High**	
**Unique ID**	2	**Study ID**	G.G. Craig, 2012	**Assessor**	**R1/R2**
**Ref or Label**		**Aim**	Adhering to intervention (the 'per-protocol' effect)	**The effect of adhering to intervention…**	Non-adherence of trial participants to their assigned intervention
**Experimental**	Silver Diamine Fluoride/potassium iodide	**Comparator**	Oxalic acid-based preparation	**Source**	Journal article(s)
**Outcome**	Reduction of pain (tooth sensitivity)	**Results**		**Weight**	1
**Domain**	**Signaling question**			**Response**	**Comments**
**Bias arising from the randomization process**	1.1 Was the allocation sequence random?			NI	The authors don't explain how the randomization was made
	1.2 Was the allocation sequence concealed until participants were enrolled and assigned to interventions?			PN	
	1.3 Did baseline differences between intervention groups suggest a problem with the randomization process?			N	
	**Risk of bias judgement**			**High**	
**Bias due to deviations from intended interventions**	2.1 Were participants aware of their assigned intervention during the trial?			PY	They didn't explain anything about taste and smell. The participants could have known. They don't explain if the interventionists knew what they were delivering.
	2.2 Were carers and people delivering the interventions aware of participants' assigned intervention during the trial?			PY	
	2.3. [If applicable:] If Y/PY/NI to 2.1 or 2.2: Were important non-protocol interventions balanced across intervention groups?			NA	
	2.4. [If applicable:] Were there failures in implementing the intervention that could have affected the outcome?			NA	

	2.5. [If applicable:] Was there non-adherence to the assigned intervention regimen that could have affected participants' outcomes?	PN	No information about non-adherence to the intervention.
	2.6. If N/PN/NI to 2.3, or Y/PY/NI to 2.4 or 2.5: Was an appropriate analysis used to estimate the effect of adhering to the intervention?	NA	
	**Risk of bias judgement**	**High**	
**Bias due to missing outcome data**	3.1 Were data for this outcome available for all, or nearly all participants randomized?	PY	
	3.2 If N/PN/NI to 3.1: Is there evidence that the result was not biased by missing outcome data?	NA	
	3.3 If N/PN to 3.2: Could missingness in the outcome depend on its true value?	NA	
	3.4 If Y/PY/NI to 3.3: Is it likely that missingness in the outcome depended on its true value?	NA	
	**Risk of bias judgement**	**Low**	
**Bias in measurement of the outcome**	4.1 Was the method of measuring the outcome inappropriate?	PN	They used the VAS scale for pain.
	4.2 Could measurement or ascertainment of the outcome have differed between intervention groups?	Y	They used the VAS scale and visual changes for the second outcome.
	4.3 Were outcome assessors aware of the intervention received by study participants?	NA	
	4.4 If Y/PY/NI to 4.3: Could assessment of the outcome have been influenced by knowledge of intervention received?	NA	
	4.5 If Y/PY/NI to 4.4: Is it likely that assessment of the outcome was influenced by knowledge of intervention received?	NA	
	**Risk of bias judgement**	**High**	
**Bias in selection of the reported result**	5.1 Were the data that produced this result in accordance with a pre-specified analysis plan that was finalized before unblinded outcome data were available for analysis?	PN	There was no information about on this topic.
	5.2 ... multiple eligible outcome measurements (e.g. scales, definitions, time points) within the outcome domain?	PY	They used VAS scoring and visual changes based on photographs
	5.3 ... multiple eligible analyses of the data?	PN	

	**Risk of bias judgement**			**High**	
**Overall bias**	**Risk of bias judgement**			**High**	
**Unique ID**	3	**Study ID**	N. Permata, 2018	**Assessor**	**R1/R2**
**Ref or Label**		**Aim**	Adhering to intervention (the 'per-protocol' effect)	**The effect of adhering to intervention…**	Non-adherence of trial participants to their assigned intervention
**Experimental**	Silver Diamine Fluoride	**Comparator**	Silver Diamine Fluoride followed by CO_2 laser treatment	**Source**	Journal article(s)
**Outcome**	Compare the efficacy of silver diamine fluoride and CO_2 laser in reducing the dentin hypersensitivity score	**Results**		**Weight**	1
**Domain**	**Signaling question**			**Response**	**Comments**
**Bias arising from the randomization process**	1.1 Was the allocation sequence random?			PY	No information on the method of randomization
	1.2 Was the allocation sequence concealed until participants were enrolled and assigned to interventions?			N	
	1.3 Did baseline differences between intervention groups suggest a problem with the randomization process?			Y	The study was not statistically significant, since some results were $p>0.05$.
	**Risk of bias judgement**			**High**	
**Bias due to deviations from intended interventions**	2.1 Were participants aware of their assigned intervention during the trial?			PY	Probably yes, because only one group applied a laser. Consequently, the participants knew which group they were from. Probably yes, because they needed to apply lasers only to one of the groups.
	2.2 Were carers and people delivering the interventions aware of participants' assigned intervention during the trial?			PY	
	2.3. [If applicable:] If Y/PY/NI to 2.1 or 2.2: Were important non-protocol interventions balanced across intervention groups?			NA	
	2.4. [If applicable:] Were there failures in implementing the intervention that could have affected the outcome?			NA	

	2.5. [If applicable:] Was there non-adherence to the assigned intervention regimen that could have affected participants' outcomes?	PN	No information on non-adherence to the intervention.
	2.6. If N/PN/NI to 2.3, or Y/PY/NI to 2.4 or 2.5: Was an appropriate analysis used to estimate the effect of adhering to the intervention?	NA	
	**Risk of bias judgement**	**High**	
**Bias due to missing outcome data**	3.1 Were data for this outcome available for all, or nearly all, participants randomized?	Y	
	3.2 If N/PN/NI to 3.1: Is there evidence that the result was not biased by missing outcome data?	NA	
	3.3 If N/PN to 3.2: Could missingness in the outcome depend on its true value?	NA	
	3.4 If Y/PY/NI to 3.3: Is it likely that missingness in the outcome depended on its true value?	NA	
	**Risk of bias judgement**	**Low**	
**Bias in measurement of the outcome**	4.1 Was the method of measuring the outcome inappropriate?	N	
	4.2 Could measurement or ascertainment of the outcome have differed between intervention groups?	N	
	4.3 Were outcome assessors aware of the intervention received by study participants?	Y	The assessors were aware.
	4.4 If Y/PY/NI to 4.3: Could assessment of the outcome have been influenced by knowledge of intervention received?	Y	Knowledge could have influenced the results
	4.5 If Y/PY/NI to 4.4: Is it likely that assessment of the outcome was influenced by knowledge of intervention received?	Y	
	**Risk of bias judgement**	**High**	
**Bias in selection of the reported result**	5.1 Were the data that produced this result analysed in accordance with a pre-specified analysis plan that was finalized before unblinded outcome data were available for analysis?	PN	There was no information on this topic.
	5.2 ... multiple eligible outcome measurements (e.g. scales, definitions, time points) within the outcome domain?	Y	They used VAS for two types of stimuli (evaporative and thermal) and also DIAGNOdent.
	5.3 ... multiple eligible analyses of the data?	PN	
	**Risk of bias judgement**	**High**	
**Overall bias**	**Risk of bias judgement**	**High**	
